# Study on the Water-Sensitivity Passivation Effect and Mechanism of PA-ES Composite Materials

**DOI:** 10.3390/ma16175872

**Published:** 2023-08-28

**Authors:** Nan Wang, Runze Wang, Qingzhao Zhang, Yuanjie Luo, Hui Xu

**Affiliations:** 1 Key Laboratory of Geotechnical and Underground Engineering of Ministry of Education, Tongji University, Shanghai 200092, China; 2 Sinopec Petroleum Engineering Design Co., Ltd., Dongying 257000, China; 3 School of Microelectronics, Shanghai University, Shanghai 200444, China

**Keywords:** polyacrylic, expansive soil, water-sensitive effect, passivator, passivation mechanism

## Abstract

The water-sensitive effect of expansive soil (ES) poses a serious challenge to the safety and durability of infrastructure. To reduce the effect of water sensitivity on expansive soil, a new powder soil passivator with polyacrylic (PA) as the main component was proposed. In this paper, a series of macroscopic and microscopic tests were conducted to evaluate the water-sensitive passivation effect and mechanism of PA-ES composites. The results showed that PA significantly attenuated the water sensitivity of ES. With the increase in PA content in the PA-ES composites, the water sensitivity of the composites decreased, swelling and shrinkage deformation decreased, and the strength of the composites increased significantly. In addition, when the content of PA in the PA-ES composite is 6%, it can significantly alleviate the deformation of the composite and improve the saturated shear strength of the composite, meeting the requirements of ES engineering disposal. Finally, the results show that the mechanism of PA passivation of ES water-sensitive effect mainly includes adsorption, binding, and filling. The study shows that PA has a broad engineering application prospect as an ES passivator.

## 1. Introduction

Expansive soils (ES) are widely distributed worldwide, in more than 40 countries on six continents [1]. They are pretty sensitive to changes in water and have significant water sensitivity effects (such as high swelling and shrinkage characteristics, and low saturation strength characteristics) [2,3,4]. In natural environments, during the rainy season, expansive soil absorbs a large amount of water and swells greatly, leading to a sudden decrease in soil strength. In the dry season, the expansive soil evaporates and the volume shrinks, resulting in soil cracks. These characteristics lead to extremely complex engineering properties, often posing challenges to infrastructure development and maintenance, such as the safe operation of major projects such as urban construction, water conservancy facilities, and highways and railways. According to the statistics, it caused more than 10 billion USD of direct economic losses per year in China [5]. Therefore, it is of great importance to study the basic characteristics of expansive soil and its treatment methods.

At present, some general understandings have been made about the engineering characteristics of expansive soil. It contains more hydrophilic clay minerals such as montmorillonite and illite, etc. [6]. These clay minerals are mainly composed of layered silicates, and the basic building blocks of clay minerals are stacked into multiple layers of tetrahedral and octahedral sheets. The framework has negatively charged tetrahedral and octahedral layers due to isostructural substitution effects [7]. These clay lamellar structures represented by montmorillonite and illite possess a strong adsorption capacity and cation-exchange capacity [8]. Under the action of the electric field force, water molecules will be adsorbed around the mineral particles to form a hydration film. The thickness change of the hydration film directly affects the swelling and shrinkage of expansive soil, which in turn affects the strength characteristics of expansive soil [9,10].

In the field of geotechnical engineering, the activation of clay minerals by water can easily lead to disasters. The properties of inorganic materials can be improved by chemical treatment [11,12]. Therefore, based on understanding its basic properties, people have conducted a lot of exploration on the passivation treatment of water sensitivity of expansive soil. Passivation treatment is the use of a passivator to react with a clay matrix, thereby reducing the ability of soil particles to interact with water and increasing soil stability and integrity. Passivators are often referred to as chemical stabilizers, and currently there are mainly traditional and non-traditional passivators [13].

The traditional passivators are mainly lime, cement, and fly ash. Since they are all calcium-based inorganic gel materials, the curing mechanism is also roughly the same; that is, an ion exchange and pozzolanic reaction through a chemical reaction between calcium oxide in the material and the surface of clay minerals. These effects reduce the thickness of the hydration film on the surface of clay minerals and increase the cohesion between soil particles, thereby reducing the swelling and shrinkage of the soil and increasing the strength of the soil [14,15]. However, the traditional passivator will generate ettringite with high expansiveness when passivating expansive soil containing sulfate, which limits the passivation effect [16,17]. It also has the shortcomings of low reactivity, obvious brittle failure, and a long construction period. In addition, the production of a traditional passivator causes a large amount of greenhouse gas, which is contributing to global climate change [18,19].

In recent years, non-traditional passivators, especially high-molecular polymers, have been increasingly used as substitutes for traditional passivators [20,21,22,23,24]. Compared with traditional passivators, they mainly have the advantages of a high reactivity, short curing period, and high environmental applicability [25,26]. At present, the polymer soil passivator is mainly based on liquid material [27,28]. The liquid soil passivator applied to clay may cause the clay to contain more water, which may have the opposite effect. Therefore, this study proposes a novel powdered soil passivator. Its main component is polyacrylic (PA), which has the advantages of easy mixing with soil particles, controllable reaction time, and strong engineering applicability.

**Scheme 1:**
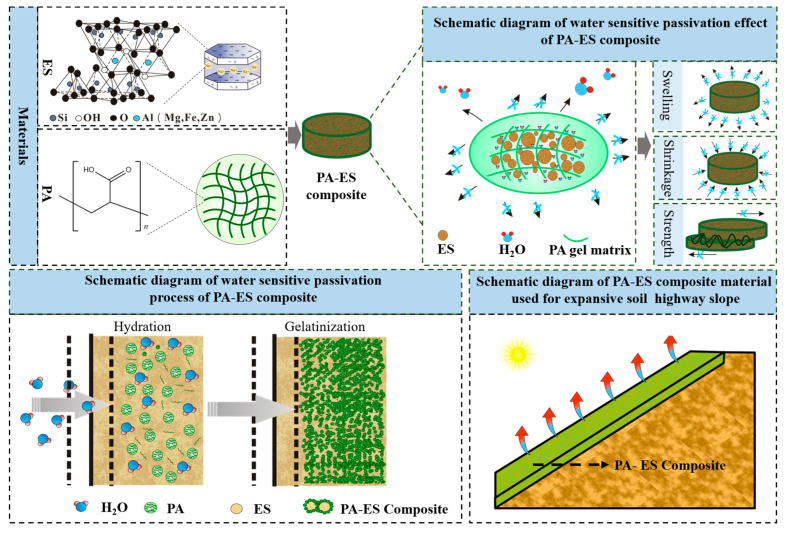
Process, principle, and application diagram of water-sensitive passivation of PA-ES composite materials.

In this study, PA was prepared in the laboratory. It contains multiple reactive groups, meaning it is easy to react with water molecules to form a flexible low permeable gel. Expansive soil particles are composed of silico-aluminate clay layers with a negative surface. Theoretically, under the action of water PA can form a gel barrier with expansive soil particles through a series of interactions (hydrogen bonding, water bridging, cation bridging, etc.) to inhibit the infiltration of water molecules into soil particles, thus slowing down soil expansion and contraction and improving soil saturation strength (Scheme 1). For this reason, the PA-ES composites were prepared by uniformly mixing PA with different contents and expansive soil. The effect of PA content on the water sensitivity of PA-ES composites was evaluated through the limit water content test. The influence of PA content on the swelling and shrinkage characteristics of the PA-ES composite was evaluated through the free swell ratio test, the no-loading swell ratio test, and the linear shrinkage ratio test, and the influence of PA content on the strength of the PA-ES composite was evaluated through the saturated direct shear test. Based on the above experiments, a comprehensive evaluation was conducted on the water-sensitive passivation effect of PA-ES composite material. In addition, the water-sensitive passivation mechanism of PA-ES composite material was analyzed through the particle distribution (PSD) test, the X-ray diffraction (XRD) test, and the environmental scanning electron microscopy (ESEM) test.

## 2. Materials and Methods

### 2.1. Materials

- ES

The ES used in this study was obtained from Anhui Province, and excavated in the open air. The collection depth was 0.5 mm at natural ground level to avoid infestation of roots, vegetation, or any organic matter. The soils were packed in plastic bags and shipped to the soil mechanics laboratory for testing. Each bag of soil was air-dried, crushed, and passed through a 0.5 mm sieve before testing. The basic physical and chemical properties of the soils were determined according to GB/T 50123-2019 [29]. Table 1 presents the corresponding results.

- PA

PA is a laboratory-prepared powdery soil passivation material that mixes with soil more easily than liquid polymer materials. It is polymerized by acrylic monomers, initiators, and corresponding additives at a certain PH, temperature, and nitrogen atmosphere. It contains a large number of active polymer chains, primarily the hydrophilic carboxyl group (-COOH) and hydroxyl group (-OH). The basic physicochemical properties of PA were determined in the laboratory. The measurement results are shown in Table 2.

- Sample preparation

In this paper, the ES was air-dried first and then passed through a 0.5 mm sieve after being crushed. To prepare a homogeneous mixture of PA and ES, the required weight of PA was first calculated from the total dry weight of ES and mixed in the dry state. The mixture of ES and PA in the dry state was then mixed with the desired water to obtain the optimum moisture content. The content of PA was 0%, 2%, 4%, 6%, and 8% of the dry weight of ES, respectively.

**Figure 1:**
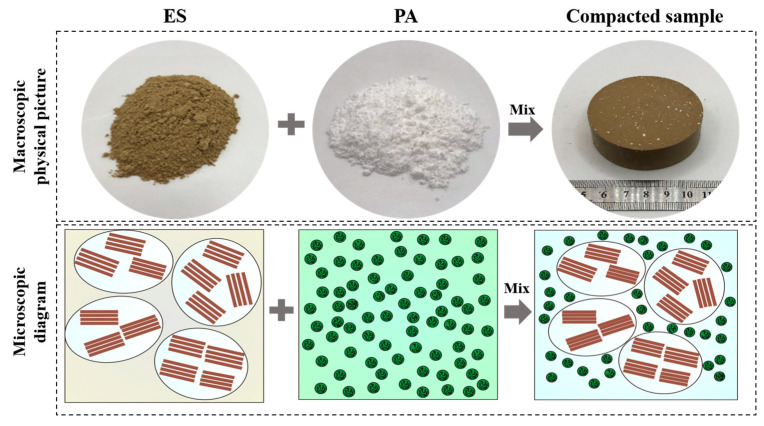
Examples of the materials that were used for testing.

According to the state of the sample, they were divided into unstructured samples and compacted samples. The unstructured samples were prepared in strict accordance with GB/T 50123-2019 [29]. Correspondingly, the compacted samples were prepared as follows: with reference to the maximum dry density and optimum water content of the soil, PA-ES mixtures with different mixing ratios were compacted into samples with a size of 61.8 mm × 20 mm (Figure 1). The compaction method adopts the static method and uses a universal testing machine to complete the entire compaction process. The compaction speed is 0.2 mm/min and the holding time is 1 h. It is important to note that after all the samples are prepared, they are kept in sealed plastic bags and cured for 24 h at the standard test temperature (20 °C) to homogenize their moisture.

### 2.2. Methods

- Water-sensitive passivation effect evaluation test

**Figure 2:**
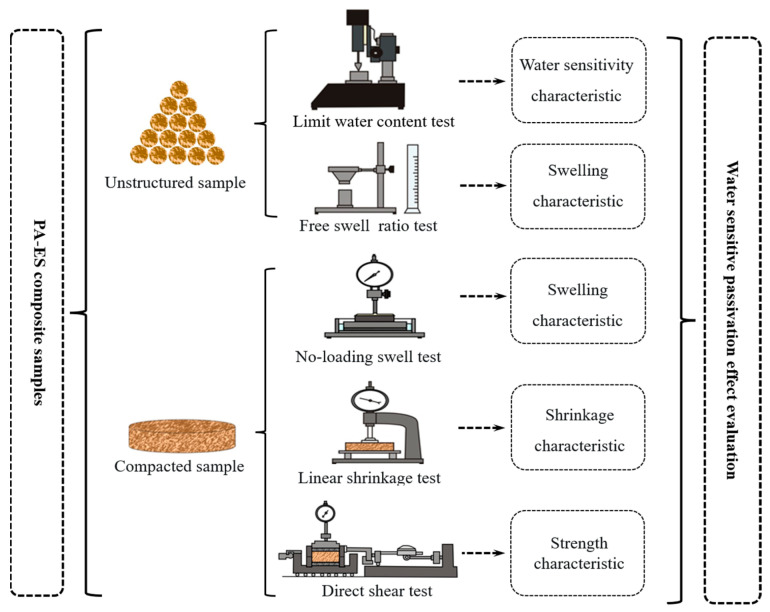
Equipment diagram of water-sensitive passivation effect evaluation tests.

Studies have shown that ES has strong water sensitivity, which leads to high swelling and shrinkage and a low saturation strength of ES, making these water-sensitive effects highly prone to disasters [9,30]. A series of laboratory tests were conducted to evaluate the effectiveness of water-sensitive passivation of PA-ES composites (Figure 2). The influence of PA content on the water sensitivity and swelling of unstructured PA-ES composites was studied by the limit water content test and the free swell ratio test. The effect of PA content on the swelling and shrinkage characteristics of the PA-ES composites was studied through the no-loading swell ratio test and linear shrinkage test. The effect of PA content on the saturation strength of the PA-ES composites was evaluated by the direct shear test. The above tests refer to GB/T 50123-2019 [29].

- Water-sensitive passivation mechanism analysis test

To clarify the mechanism of PA on water-sensitive deactivation of ES, two groups of PA-ES composite containing 0% PA and 6% PA were tested by the PSD test, the XRD test, and the ESEM test. The PSD test can provide information on the particle size distribution and clay content of the sample, the XRD test can help observe changes in mineral and crystal phases of the sample, and the ESEM test can provide a qualitative evaluation of the microstructure of the sample.

For this purpose, a Malvern Mastersizer 2000 from Malvern in the United Kingdom was used for the PSD test. The dry powdery samples containing 0% PA and 6% PA were first dispersed into a non-reactive liquid and then sent to the system for analysis. After analysis, parameters such as particle size were determined by particle size distribution and statistical spreadsheet programs. An X-ray diffractometer (PANalytical X’Pert3 Powder) was used for the XRD test. Dried samples (0%PA, 6%PA) were ground to a fine powder in an agate mortar and sieved, then subjected to XRD analysis using Cu Ka radiation on a PANalytical X’Pert3 Powder instrument. Identification of crystal phases and peaks was performed using JADE 6.5 software. ESEM (Quanta 400 from FEI) was used to study the microstructure of samples containing 0% PA and 6% PA; images were collected on freshly fractured surfaces.

## 3. Results

### 3.1. Water-Sensitive Passivation Effect Evaluation Test

- Limit water content test

**Figure 3:**
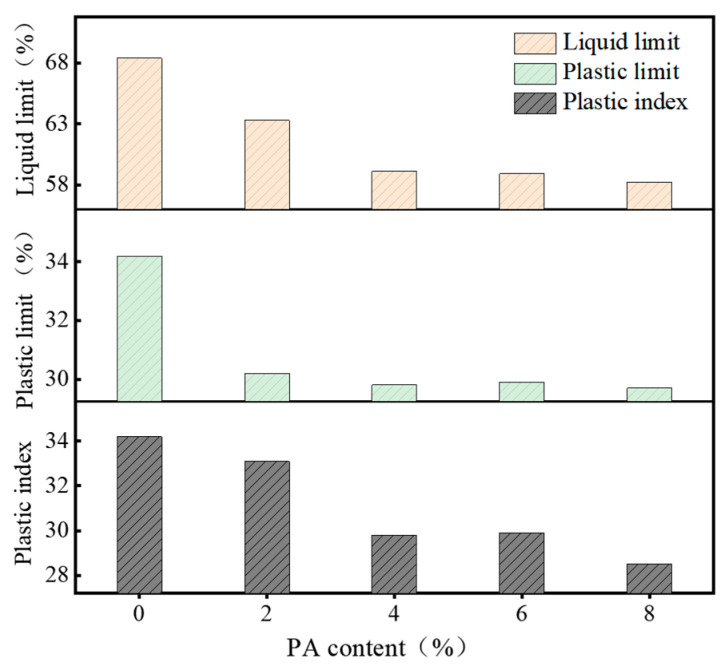
Limiting water content test results for PA-ES composites with different PA contents.

Figure 3 shows the results of the limit water contents of PA-ES composites with different PA contents. It can be seen that as the content of PA increases, the liquid limit (LL), plastic limit (PL), and plastic index (PI) of ES decreases. With an increase in the PA content from 0% to 8%, the LL decreased from 68.4% to 58.2%, while the PL decreased from 34.2% to 29.7%. When PA was added at 0–4%, the PI (PI = LL − PL) decreased significantly, from 34.2% to 29.8%. When the PA addition amount is 4–8%, the plasticity index change is not obvious. Therefore, the increase in PA content significantly reduces the limit water content and passivates the water sensitivity of the PA-ES composites.

- Free swell ratio test

**Figure 4:**
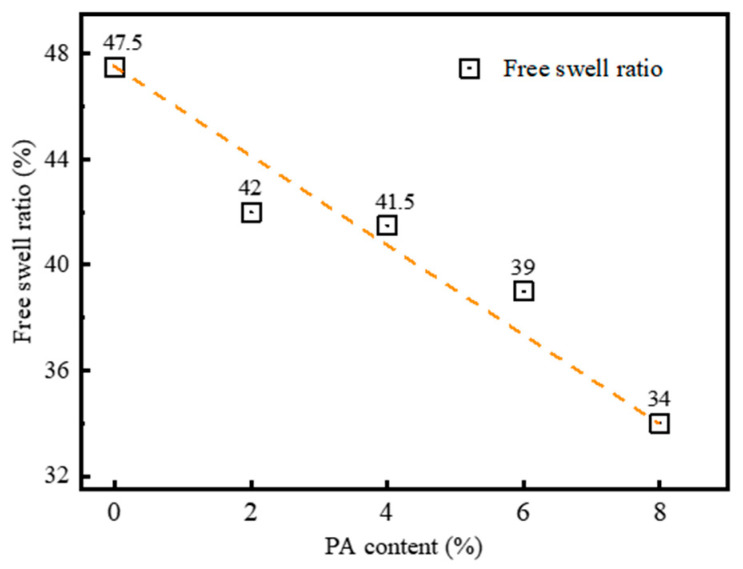
Free swell ratio test results for PA-ES composites with different PA contents.

The results of the free swell ratio test are shown in Figure 4. The results show that when PA content increases from 0% to 8%, the free swell ratio decreases from 47.5% to 34%; in particular, when PA content reaches 6%, the free swell ratio decreases to 39%. According to GB 50112-2013 [31], the water-sensitive effect of samples containing 6% PA is no longer significant and does not belong to the category of expansive soil. In general, the relationship between the free swell ratio and PA content shows a linear decline, which is similar to the results of previous studies [32,33].

- No-loading swell ratio test

**Figure 5:**
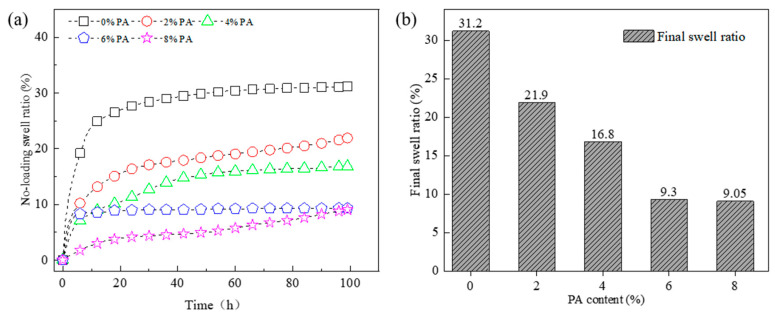
No-loading swell ratio test results for PA-ES composites: (**a**) the relationship between no-loading swell ratio and time history under different PA contents, (**b**) the relationship between PA content and final no-loading swell ratio.

Figure 5a illustrates the changes in the no-loading swell ratio over time for PA-ES composites with different PA contents. The no-loading swell ratio of samples was largely reduced by PA. Generally, the no-loading swell ratio developed rapidly at the 0 to 10th hours and then gradually stabilized over time for samples with a PA content of 0~6%. Both the changing ratio and stabilized value were lower for higher PA contents. However, when the PA content of the sample was 8%, the no-loading swell ratio increased abnormally after 20~40 h, accompanied by the phenomenon of gel overflow on the soil surface. Figure 5b shows the relationship between the final no-loading swell ratio of the PA-ES composites and the PA content. With the PA content increasing from 0% to 8%, the no-loading swell ratio decreased from 31.2% to 9.05%. When the content of PA was 2%, 4%, and 6%, the final swell ratio was 21.9%, 16.8%, and 9.3%, respectively. The above phenomenon indicates that the increase in PA content in the PA-ES composites is helpful for inhibiting the swelling of samples, especially when the PA content is 6%.

- Linear shrinkage test

**Figure 6:**
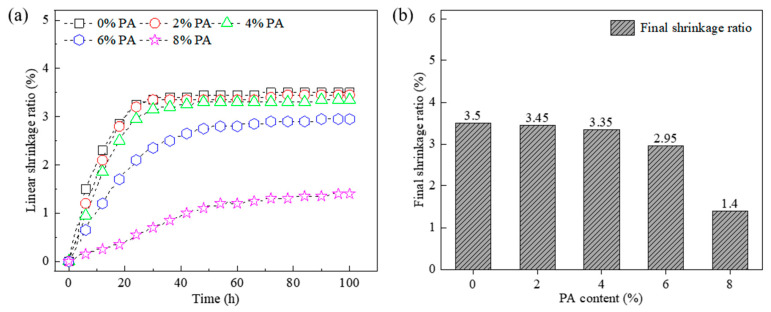
Linear shrinkage test results for PA-ES composites: (**a**) the relationship between linear shrinkage ratio and time history under different PA contents, (**b**) the relationship between PA content and final shrinkage ratio.

The linear shrinkage ratio of PA-ES composites with different PA contents varies with drying time, as shown in Figure 6a. The linear shrinkage of all samples increases during the initial drying stage and gradually stabilizes with increasing drying time. As the PA content in the sample increases, the linear shrinkage ratio significantly decreases. Figure 6b shows the relationship between the final shrinkage ratio and the PA content of the PA-ES composites. It can be found that compared with PA-ES composites with a PA content of 1–4%, PA-ES composites with a PA content of 6–8% have a more significant final shrinkage inhibition effect: when the PA content is 0–4%, the final shrinkage ratios are 3.5% and 3.35%, respectively; when the PA content is 6–8%, the final shrinkage ratios significantly decrease, reaching 2.95% and 1.4%, respectively. This indicates that the increase in PA content has a good inhibitory effect on the contraction of the PA-ES composite.

- Direct shear test

**Figure 7:**
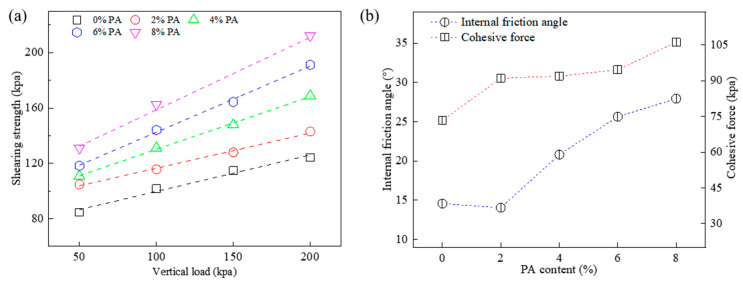
Direct shear strength test results for PA-ES composites: (**a**) the relationship between shear strength and vertical load under different PA contents, (**b**) the relationship between PA content and shear strength parameters.

The direct shear test results of PA-ES composites under different vertical loads are shown in Figure 5. As shown in Figure 7a, the saturated shear strength of the PA-ES composites increases with the increase in vertical load and PA content. When the vertical loads were 50 kPa, 100 kPa, 150 kPa, and 200 kPa, the saturated shear strength of the PA-ES composites increased by 54.93%, 60.02%, 57.75%, and 71.09%, respectively, with the addition of PA increasing from 0% to 8%. In other words, the PA content and vertical pressure in the PA-ES composite have positive effects on the saturated shear strength.

The saturated shear strength parameters of the PA-ES composites with different PA contents were obtained by linear fitting of the shear strength under different vertical loads. As shown in Figure 7b, with the increase in PA content in the PA-ES composites, the saturated shear strength parameters of the sample change specifically manifested as an increase in the cohesion and internal friction angle of the sample. When the PA content increased to 2%, the cohesion force increased by 24.18%, and the internal friction angle did not change much. Therefore, when PA is added to 2%, the increase in saturated shear strength of the sample is mainly due to the cohesion between sample particles. When the PA content is 2% to 6%, the maximum internal friction angle increases by 75.98%, and the cohesion force increases slightly. The increase in sample shear strength is mainly due to the friction between sample particles when the content of PA is 2~6%. When the PA content is 6% to 8%, the maximum cohesion force increases by 44.77% and the maximum internal friction angle increases by 91.63%. In this case, the increase in shear strength is determined by cohesion action and internal friction action. To summarize, the addition of PA largely improved the shear strength of the PA-ES composite.

### 3.2. Water-Sensitive Passivation Mechanism Analysis Test

According to the results of the limit water content test, free swell ratio test, no-loading swell test, linear shrinkage test, and direct shear test, and considering economic factors, the optimum content of PA for the PA-ES composite studied was around 6%. To explore the action mechanism of water-sensitive passivation of the PA-ES composite from the viewpoint of a microscopic point, a series of experiments were carried out including particle size, mineral composition, and microscopic imaging using the PSD test, the XRD test, and the ESEM test.

- PSD test

**Figure 8:**
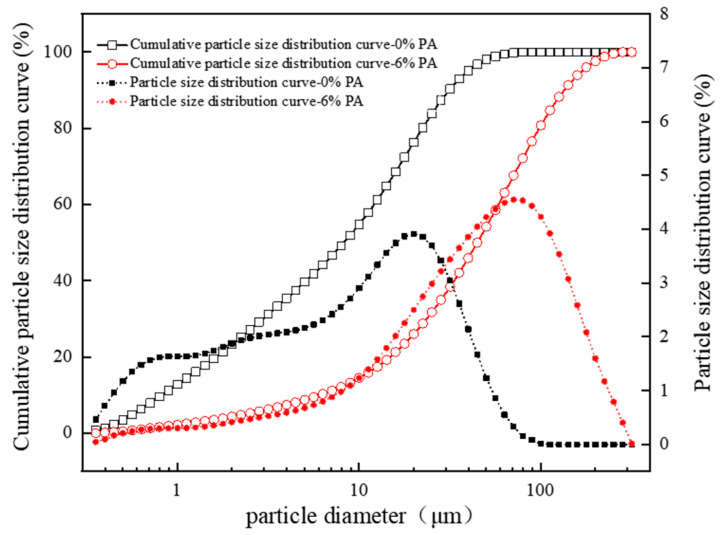
Particle size distribution of PA-ES composites with 0% and 6% PA content.

The particle size distribution of PA-ES composites with 0% PA and 6% PA content was studied, and the results are shown in Figure 8. Compared with the sample with 0% PA content, the clay content (D ≤ 0.002 mm) of the sample containing 6% PA decreased from 23.24% to 4.34%, indicating a decrease in the number of fine particles and an increase in the number of coarse particles. Based on the test results of the particle size distribution test, the grading index of the non-uniformity coefficient (Cu) and curvature coefficient (Cs) was calculated by GB/T 50123-2019 (Geotechnical Engineering Test Method Standard 2019). Compared with the sample with 0% PA content, the non-uniformity coefficient (Cu) of the sample containing 6% PA decreased from 15.85 to 8.91, and the curvature coefficient (Cs) increased from 0.79 to 1.41. It can be seen that PA can optimize the particle distribution of the sample.

- XRD test

**Figure 9:**
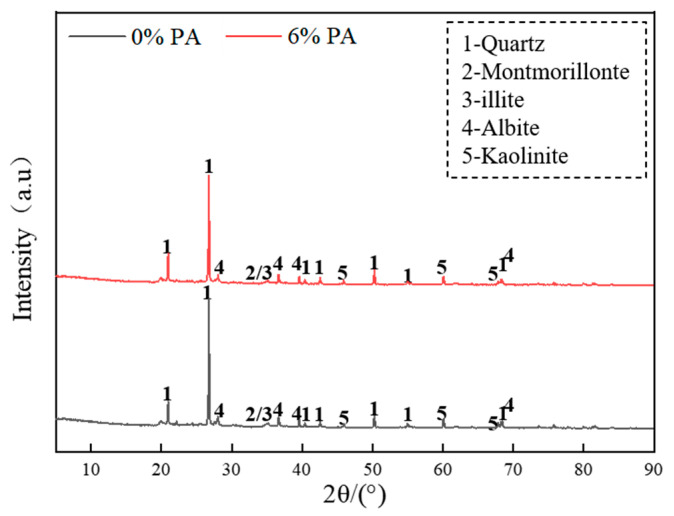
XRD of PA-ES composites with 0% and 6% PA content.

Figure 9 shows the XRD pattern of PA-ES composites containing 0%PA and 6%PA. As can be seen from Figure 9, the sample containing 0%PA contains quartz, montmorillonite, illite, kaolinite, and albite. The main reason for expansive soil is the presence of clay minerals such as montmorillonite, illite, and kaolinite in the soil. These clay minerals have two-dimensional layered structures, strong water absorption, and structural instability. As shown in Figure 9, there is no change in mineral composition in the sample with a PA of 6% compared to the sample with a PA of 0%. This suggests that the water-sensitive passivation mechanism of the PA-ES composite is not achieved by the formation of new minerals.

- ESEM test

**Figure 10:**
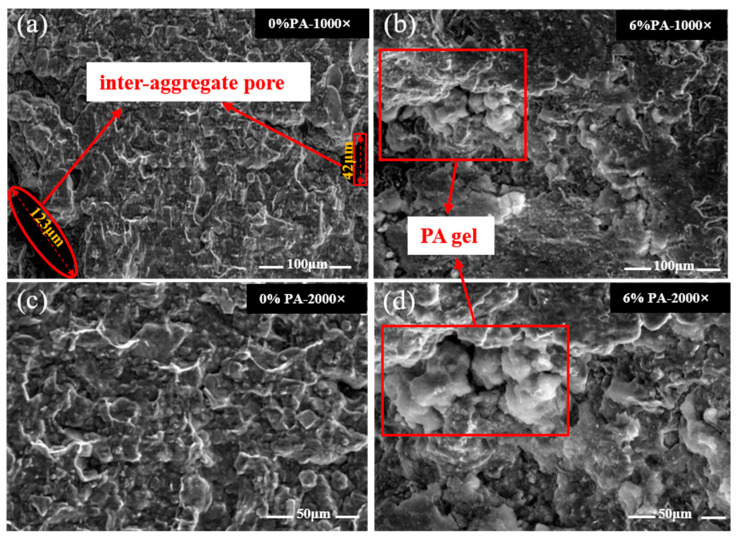
The ESEM images of PA-ES composites with (**a**) 0% PA at ×1000 magnification, (**b**) 6% PA at ×1000 magnification, (**c**) 0% PA at ×2000 magnification, (**d**) 6% PA at ×2000 magnification.

As shown in Figure 10, we studied the micromorphologies of samples containing 0%PA and 6%PA at different multiples. Figure 10a,c show the microstructure of the PA-ES composite containing 0%PA. There are large pores in the image, which may threaten the stability of the structure. Figure 10b,d show microscopic images of the PA-ES composite containing 6%PA. As can be seen from the figure, PA and ES can combine well, fill the pores between aggregates in the structure, wrap ES particles, and play a good bonding effect.

## 4. Discussion

Considering the advantages of polymer materials such as a high reactivity, short curing period, and strong environmental applicability [25,26], PA was selected as the passivation agent for ES. A series of tests of PA-ES composites were carried out to evaluate the water-sensitive passivation effect of PA on ES and its mechanism. The addition of PA greatly improves the engineering performance of soil mass, which is mainly manifested in the decrease in water sensitivity, the decrease in expansion and contraction, and the increase in strength. The soil samples in the limit water content test and free swell ratio test are all unstructured. The experimental results show that when the content of PA is 2–4%, the limit water content and free swell ratio change obviously, but when the content of PA is 4–8%, the limit water content and free swell ratio change only slightly. The results indicate that PA can improve the sample particle diameter significantly when added with 2~4% PA. The results show that the addition of 2~4% PA has a significant effect on the sample particles. This phenomenon is due to the flocculation of PA and ES under the action of water, and that the clay particles are easy to further bond into larger particle sizes, which reduces the water sensitivity and inhibits the swelling of soil particles. Tan et al. [34] and Yazdandoust et al. [35] also confirmed that polymers can increase the size of clay particles through flocculation and bonding, thereby reducing the sample limit water content and inhibiting swelling.

Different from the unstructured sample, the compacted structural sample is mainly composed of grain skeleton and pores. The compacted state provides constraint conditions for sample deformation and gives the sample a certain strength. The samples used in the swelling and shrinkage tests and saturated direct shear tests are both compacted samples. The results show that, with the increase in PA content, the swelling and shrinkage of the compacted samples are significantly weakened, and the shear strength is greatly increased. Adding too much PA can have adverse effects; it is worth noting that when PA content in the PA-ES composite was increased to 8%, an abnormal gel phenomenon appeared on the soil surface. Therefore, adding more PA is not always better. Soltani et al. [36] explained this phenomenon in their earlier study, which showed that when the polymer in soil exceeded the “maximum flocculation dosage”, it would self-bind into aggregates, which would produce adverse effects. Therefore, increasing the content of the passivator in the soil does not necessarily make it better. A similar conclusion has been reached by different researchers using different materials [37,38,39].

Cohesion can reflect a change in the interaction between sample particles, and the internal friction angle can reflect a change in the friction force and bite force between sample particles [40,41]. When the PA content is 2%, the cohesion of the sample increases significantly, while the internal friction angle changes slightly, indicating that the increase in shear strength is related to the change in sample particle properties. This is also in accordance with the test results of limiting water content and free swelling ratio. When the PA was 4~8%, the internal friction angle began to increase significantly, which indicated that the bite force and friction force between sample particles increased. It can be seen that when PA content in the sample is low (PA < 4%), the effect of PA is mainly reflected in the properties of sample particles, which is mainly manifested as the decrease in reactivity of sample particles, the increase in cohesivity between sample particles, and the increase in sample particle diameters, which corresponds to the results of PSD test. When the content of PA is high (4~8%), the effect of PA is not only reflected in changes in the properties of sample particles but also in an increase in the structural integrity of the sample, which is specifically manifested in the connection filling between pore structures. This corresponds to the results of the ESEM test. Liu et al. [42] studied the influence of STW organic polymer materials on the strength of soil. The results showed that the increase in soil strength was achieved through two mechanisms: polymer-filling the soil pores and chemical reactions occurring between the polymer and soil particles. Liu et al. [43] also investigated the effects of polymer materials on the shear strength of clay and found that polymer hydrogels can increase the internal friction angle and cohesion of clay samples, which is consistent with the results of this experiment.

**Figure 11:**
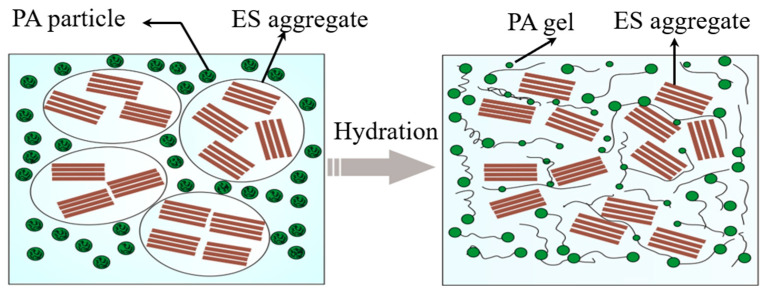
Mechanism diagram of water-sensitive passivation effect of PA-ES composite.

Based on the above discussion, a mechanism diagram of the water-sensitive passivation effect of the PA-ES composite was drawn (Figure 11). There are a considerable number of organic reactive groups in the PA polymer chains, which are uniformly pressed into the pore structure of the sample. When the PA meets water, the polymer chain unfolds gradually and the reaction is activated. With the progress of hydration, on the one hand, the surface of ES particles reacts with some active groups in PA. The surface reactivity of ES particles is changed through adsorption and bonding, and the size of ES particles is increased. On the other hand, as PA further cemented, adhered to, and filled the occluded sample pore structure, a three-dimensional gel structure was finally formed in the sample. The engineering function of this three-dimensional gel structure can be expressed as follows. When absorbing water and wetting, it effectively limits the water entering into the ES particles, increases the toughness and strength of the sample after saturation in water, and can flexibly offset the swelling deformation. When losing water and drying, the gel barrier formed between the particle will limit the discharge of water from the sample, locking the water inside the ES particles and limiting the shrinkage of the sample.

## 5. Conclusions

To study the potential application of polyacrylic (PA) as an expansive soil (ES) passivator in engineering, the water-sensitive passivation effects of the PA-ES composite, such as the limit water content, free swell ratio, no-load swell ratio, linear shrinkage ratio, and direct shear strength, were tested and evaluated. At the same time, particle size distribution characteristics, mineral composition characteristics, and microscopic imaging characteristics of samples before and after PA passivation treatment of ES were tested to clarify the mechanism of water-sensitive passivation of the PA-ES composite. The main conclusions are as follows:

(1) PA can improve the physical properties of the PA-ES composite. The specific performance is that the liquid limit, plastic limit, and plasticity index all decrease with the increase in PA content, and the interaction activity with water decreases.
(2) PA can effectively inhibit the deformation of the PA-ES composite. With the increase in PA content, both the swelling index and the shrinkage index show a downward trend. When the PA content is 6%, the deformation of the sample can be effectively suppressed.
(3) PA can significantly improve the mechanical properties of the PA-ES composite. Specifically, the saturated shear strength, cohesion, and internal friction angle are significantly increased.
(4) When the PA-ES composite reacts with water, PA increases the ES particle size through adsorption, reduces the content of clay particles, optimizes the gradation, and thus reduces the water reactivity of clay particles. By bonding, agglomeration, and filling, the deformation of the composite was inhibited and the strength of the composite was improved. The above reaction mechanism is also reflected in the macroscopic engineering characteristics of the PA-ES composite.
(5) PA is a promising soil passivator. When PA content is 6%, the PA-ES composite has a good water-sensitive passivation effect, which can provide an engineering reference for the treatment of expansive soil.

## Figures and Tables

**Table 1:** Basic physical properties of ES.

Soil Properties	Value
Nature water content (%)	26.56
Dry unit weight (g/cm^3^)	1.51
Liquid limit (%)	63.8
Plastic limit (%)	30.2
Free swell ratio (%)	47.8%
Optimum moisture content (%)	17%
Maximum dry unit weight (g/cm^3^)	1.76
Specific gravity	2.71
Passing 0.05 mm sieve (%)	41.13%

**Table 2:** Basic properties of PA.

Characteristics	PA
Molecular formula	(C_3_H_4_O_2_)_n_
Molecular weight *M_w_*	1.30 × 10^5^
Mean grain size (μm)	53
PH	6.9
State	Powder
Color	White
Solvability in Water	Solution
Unit weight (g/cm^3^)	1.29
Solid content (%)	46.6%

## Data Availability

Data are contained within the article.
